# Occupational medicine and toxicology

**DOI:** 10.1186/1745-6673-1-1

**Published:** 2006-02-02

**Authors:** David A Groneberg, Axel Fischer

**Affiliations:** 1 Allergy-Centre-Charité & Institute of Occupational Medicin, Charité – Universitätsmedizin Berlin, Free University and Humboldt University, Augustenburger Platz 1 OR-1, D-13353 Berlin, Germany

## Abstract

This editorial is to announce the *Journal of Occupational Medicine and Toxicology*, a new Open Access, peer-reviewed, online journal published by BioMed Central. Occupational medicine and toxicology belong to the most wide ranging disciplines of all medical specialties. The field is devoted to the diagnosis, prevention, management and scientific analysis of diseases from the fields of occupational and environmental medicine and toxicology. It also covers the promotion of occupational and environmental health. The complexity of modern industrial processes has dramatically changed over the past years and today's areas include effects of atmospheric pollution, carcinogenesis, biological monitoring, ergonomics, epidemiology, product safety and health promotion. We hope that the launch of the *Journal of Occupational Medicine and Toxicology *will aid in the advance of these important areas of research bringing together multi-disciplinary research findings.

## Editorial

The *Journal of Occupational Medicine and Toxicology *is an Open Access, online journal that publishes research articles, short reports and reviews related to the field of occupational/environmental medicine and toxicology. The first published articles focus on respiratory medicine and infectious diseases [1-5].

## The field of research

Occupational medicine and toxicology belongs to the most wide ranging disciplines of all medical specialties. The field is devoted to the diagnosis, prevention, management and scientific analysis of occupational diseases, injuries and disability. It also covers the promotion of health of workers, their families, and communities. In the era of heavy industries occupational medicine was known as "industrial medicine" with a focus on acute medical care for injured employees. The complexity of modern industrial processes changed the field and today's areas covered by occupational medicine include effects of atmospheric pollution, carcinogenesis, biological monitoring, ergonomics, epidemiology, product safety and health promotion.

## Why the journal is needed

The field of occupational medicine and toxicology is not static and the demand for studies addressing the large variety of current issues continues to grow. In view of this demand and the fact that numerous occupational medicine and toxicology studies are published by journals of related fields such as pulmonary medicine, oncology or allergy, there is a need for a new online journal that also aims to improve communication between basic and clinical science. These features will be combined by an "Open Access model " and a rapid publication process without major space limitations.

## The Open Access Policy

The *Journal of Occupational Medicine and Toxicology's *Open Access policy changes the way in which articles are published. First, all articles become freely and universally accessible online, and so an author's work can be read by anyone at no cost. Second, the authors hold copyright for their work and grant anyone the right to reproduce and disseminate the article, provided that it is correctly cited and no errors are introduced [6]. Third, a copy of the full text of each Open Access article is permanently archived in an online repository separate from the journal. The *Journal of Occupational Medicine and Toxicology's *articles are archived in PubMed Central [7], the US National Library of Medicine's full-text repository of life science literature, and also in repositories at the University of Potsdam [8] in Germany, at INIST [9] in France and in e-Depot [10], the National Library of the Netherlands' digital archive of all electronic publications. Open Access has four broad benefits for science and the general public. First, authors are assured that their work is disseminated to the widest possible audience, given that there are no barriers to access their work. This is accentuated by the authors being free to reproduce and distribute their work, for example by placing it on their institution's website. It has been suggested that free online articles are more highly cited because of their easier availability [11]. Second, the information available to researchers will not be limited by their library's budget, and the widespread availability of articles will enhance literature searching [12]. Third, the results of publicly funded research will be accessible to all taxpayers and not just those with access to a library with a subscription. As such, Open Access could help to increase public interest in, and support of, research. Fourth, a country's economy will not influence its scientists' ability to access articles because resource-poor countries (and institutions) will be able to read the same material as wealthier ones (although creating access to the internet is another matter [13]).

## Peer reviewing

The journal's peer review policy covers is characterized by a "voluntarily open peer review" process. Submitted articles will generally be reviewed by two experts with the aim of reaching a decision as soon as possible. Reviewers should sign their reports according to the voluntarily open peer review policy but are not obliged to do so. They are asked to declare any competing interests.

## Competing interests

The Editors-in-Chief and the Editorial Board of the *Journal of Occupational Medicine and Toxicology *have no financial incentive to accept manuscripts as they are not paid on the basis of the number of manuscripts accepted. In fact, they do not receive any financial remuneration for their involvement with the journal. We insist that decisions about a manuscript are based on the quality of the work, not on whether the authors' can pay the article-processing charge. Authors will be asked to declare any competing interests that they have.

## Conclusion

Bringing together multi-disciplinary research findings of the field, we hope that the launch of the *Journal of Occupational Medicine and Toxicology *will aid in the advance of these important areas of research.
